# Supplementary material for: Consecutive Prostate Cancer Specimens Revealed Increased Aldo–Keto Reductase Family 1 Member C3 Expression with Progression to Castration-Resistant Prostate Cancer
Source: J Clin Med. 2019 May 1;8(5):601. doi: 10.3390/jcm8050601 (PMC6571723; doi:10.3390/jcm8050601)
Supplement: Supplementary file 1 [file jcm-08-00601-s001.pdf]

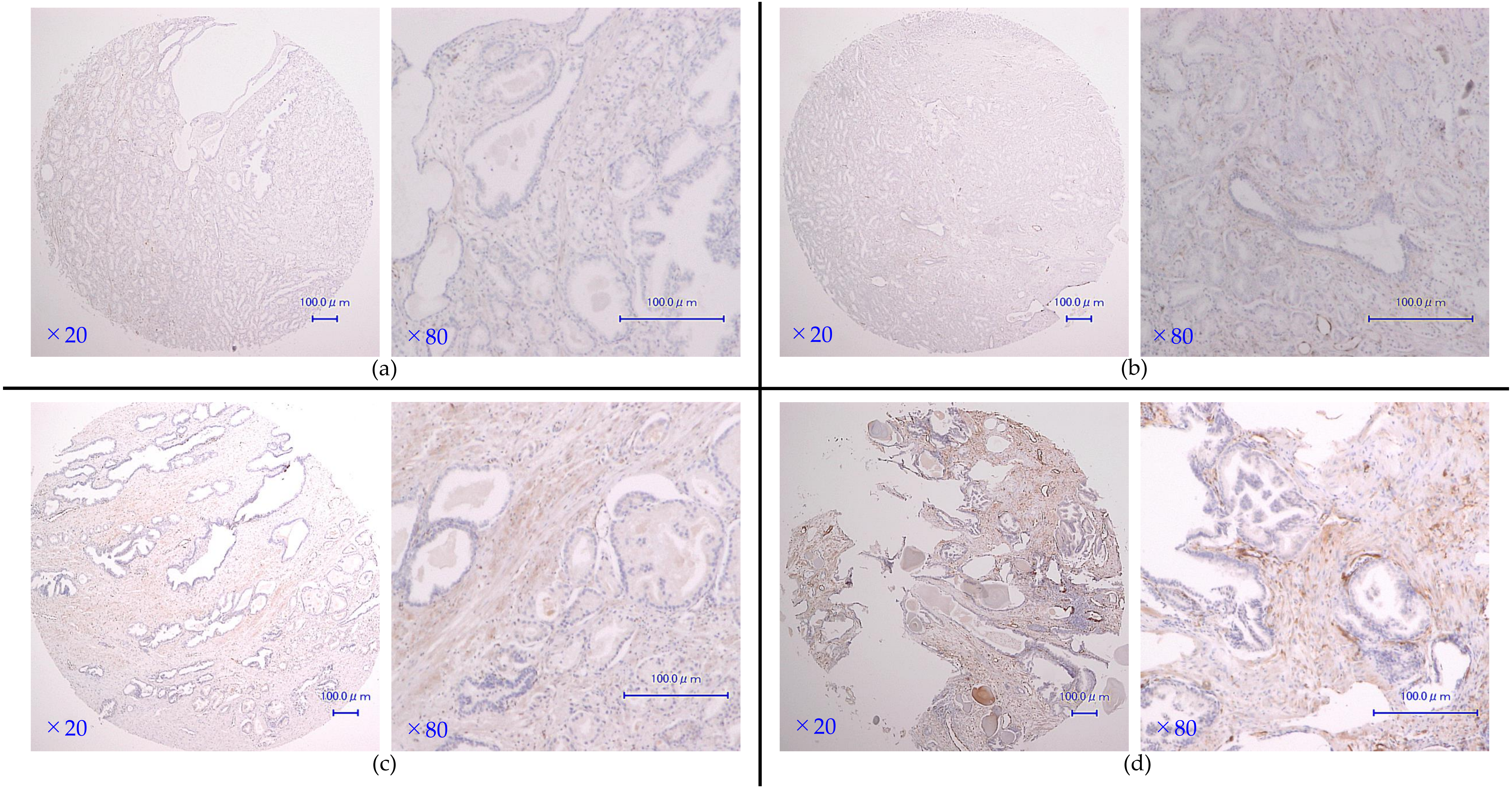

Figure S1. Representative immunostainings of AKR1C3. (**a**) benign epithelium: Score 0, cancer epithelium: Score 0, (**b**) benign epithelium: Score 1, cancer epithelium: Score 0, (**c**) benign epithelium: Score 2, cancer epithelium: Score 0, (**d**) benign epithelium: Score 3, cancer epithelium: Score 0

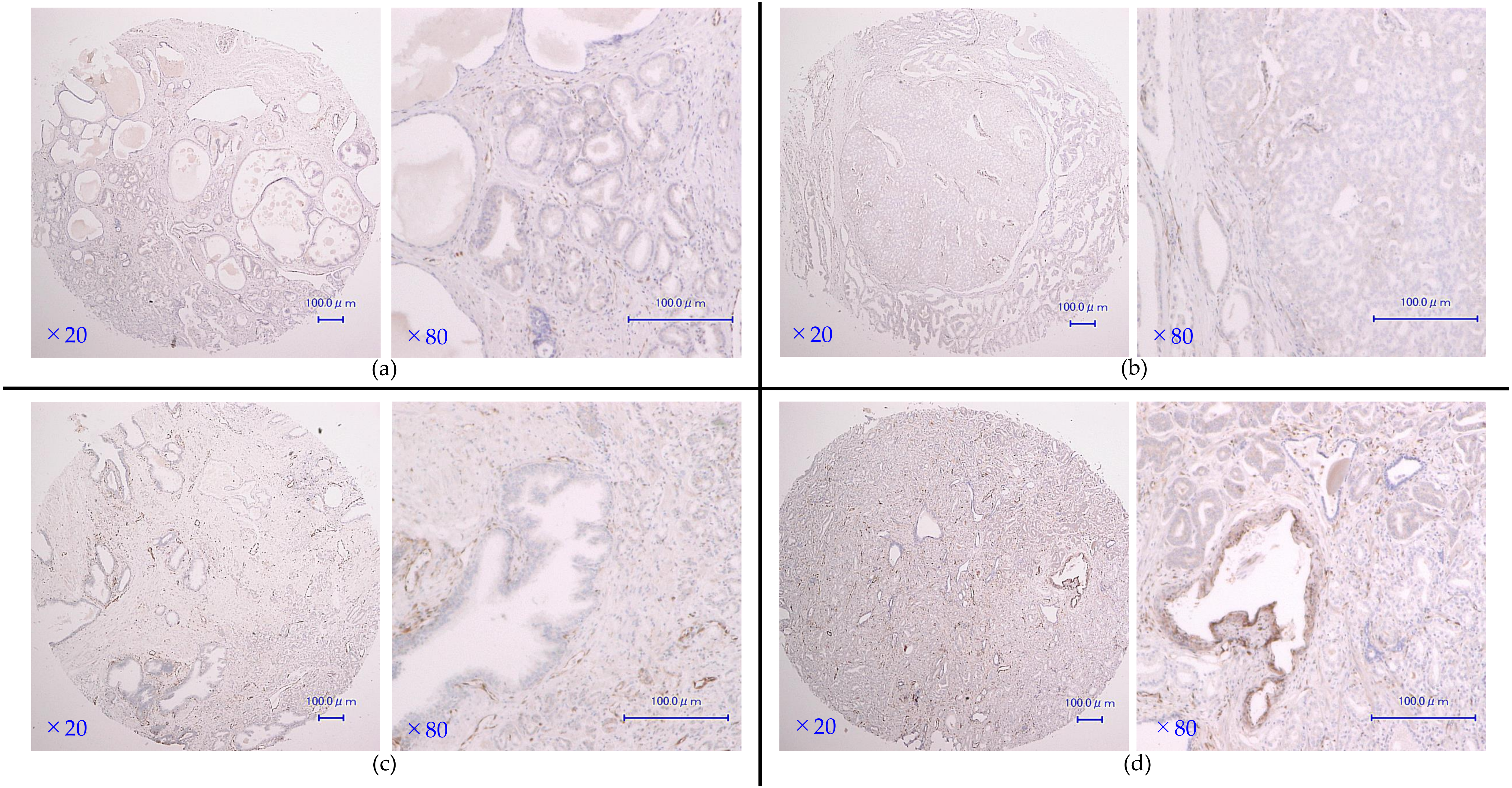

Figure S2. Representative immunostainings of AKR1C3. (**a**) benign epithelium: Score 0, cancer epithelium: Score 1, (**b**) benign epithelium: Score 1, cancer epithelium: Score 1, (**c**) benign epithelium: Score 2, cancer epithelium: Score 1, (**d**) benign epithelium: Score 3, cancer epithelium: Score 1

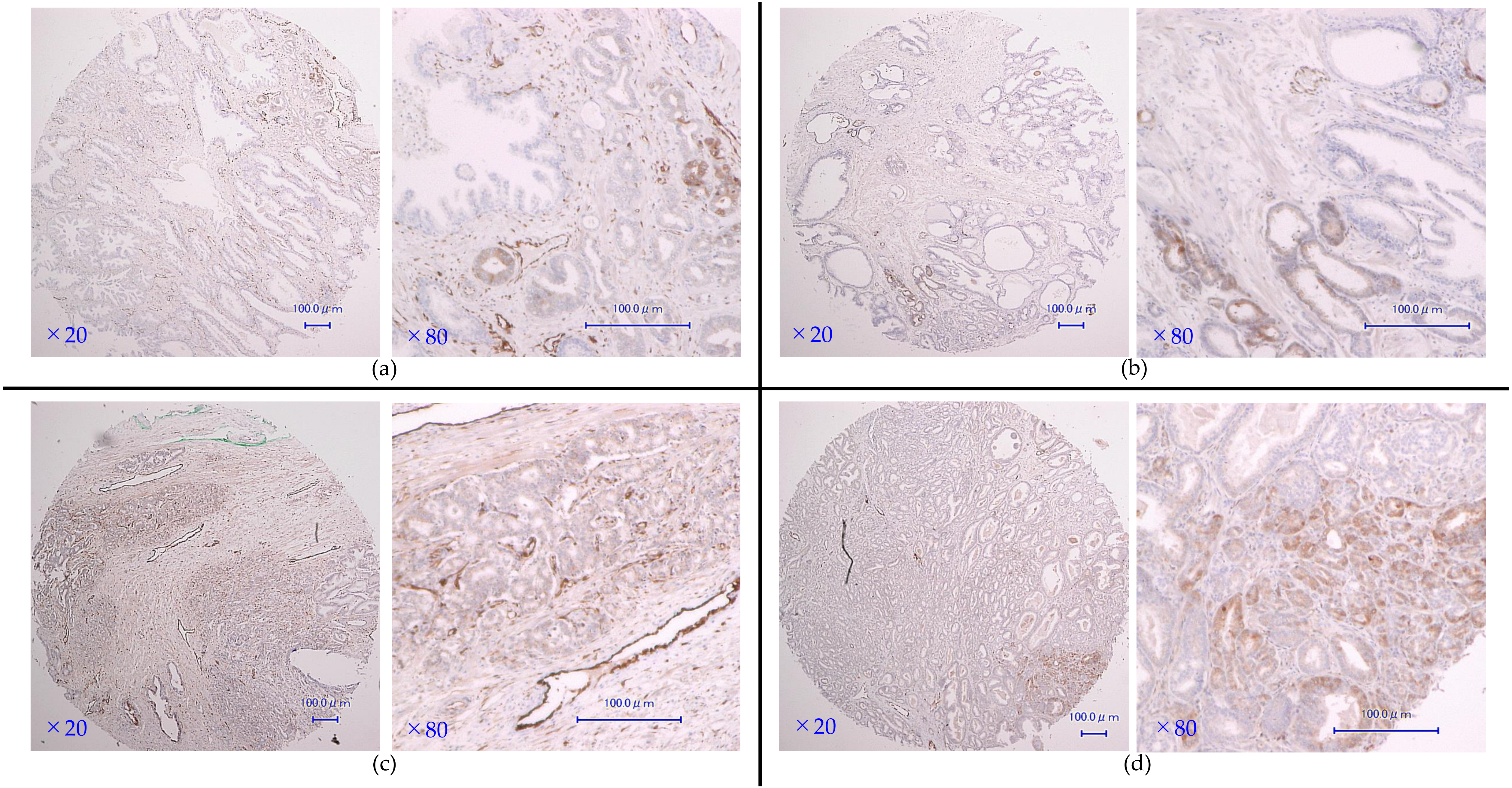

Figure S3. Representative immunostainings of AKR1C3. (**a**) benign epithelium: Score 0, cancer epithelium: Score 2, (**b**) benign epithelium: Score 1, cancer epithelium: Score 2, (**c**) benign epithelium: Score 2, cancer epithelium: Score 2, (**d**) benign epithelium: Score 3, cancer epithelium: Score 2

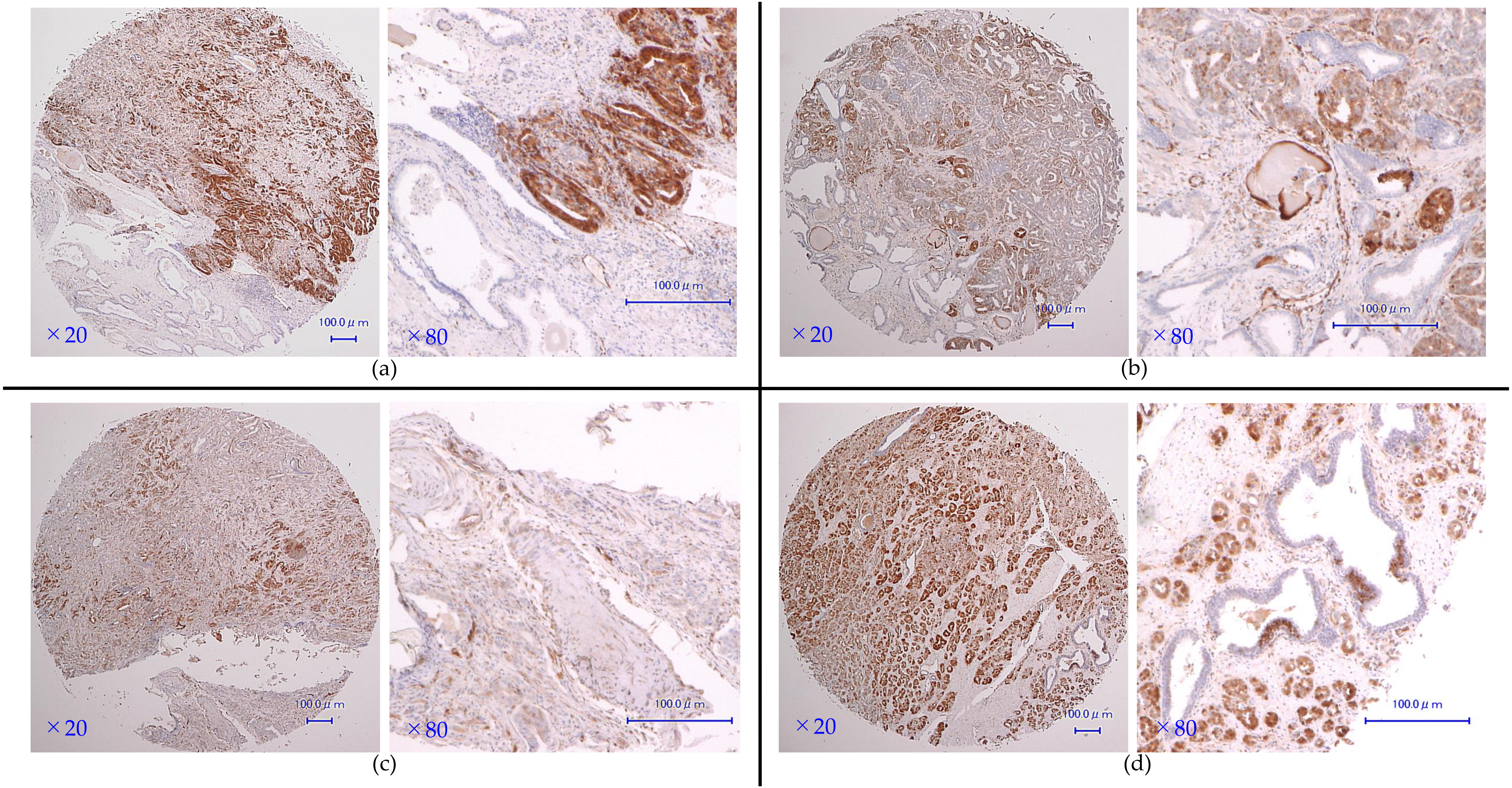

Figure S4. Representative immunostainings of AKR1C3. (**a**) benign epithelium: Score 0, cancer epithelium: Score 3, (**b**) benign epithelium: Score 1, cancer epithelium: Score 3, (**c**) benign epithelium: Score 2, cancer epithelium: Score 3, (**d**) benign epithelium: Score 3, cancer epithelium: Score 3
